# SOX combined with intraperitoneal perfusion of docetaxel compared with DOS regimen in the first-line therapy for advanced gastric cancer with malignant ascites: a prospective observation

**DOI:** 10.1186/s13063-022-06143-w

**Published:** 2022-03-12

**Authors:** Yehong Bin, Dong Lan, Wenguang Bao, Haiyan Yang, Shengsheng Zhou, Fengxiang Huang, Man Wang, Zhigang Peng

**Affiliations:** Department of Oncology, the First Affiliated Hospital of Guangxi Medical University, No.6 Shuangyong Road, Nanning, 530021 China

**Keywords:** Advanced gastric cancer, Malignant ascites, Intraperitoneal infusion of chemotherapy, First-line chemotherapy, Docetaxel, S-1, Oxaliplatin

## Abstract

**Objective:**

This study aimed to verify the survival superiority of the combination of intraperitoneal perfusion and systemic chemotherapy over standard systemic chemotherapy.

**Methods:**

A total of 78 advanced gastric cancer patients with malignant ascites were randomly divided into D-SOX group (intraperitoneal infusion of docetaxel 30 mg/m^2^ on d1 and d8, intravenous oxaliplatin 100 mg/m^2^ on d1, and oral administration of S-1 on d1-d14) and DOS group (intravenous docetaxel 60 mg/m^2^ on d1, intravenous oxaliplatin 100 mg/m^2^ on d1, and oral administration of S-1 on d1-d14). Efficacy of both groups was evaluated every 2 cycles with 21 days as a cycle. The primary endpoint was overall survival, and the secondary endpoints were objective response rate, ascites control rate, negative conversion rate of ascites cytology, and side effects.

**Results:**

The median overall survival in D-SOX group was significantly higher than that in the DOS group (11.7 vs 10.3 months, HR 0.52, 95%CI 0.31–0.86, *P* = 0.005). The ascites control rate in the D-SOX group was 58.9% and 30.8% in DOS group (95%CI 42.8–75.1% vs 95%CI 15.6–45.9%, *P* = 0.012). Besides, the adverse reactions were tolerable in both groups, and patients in the D-SOX group had lower grade 3/4 blood toxicity than that in the DOS group (26% vs 54%, *P* = 0.01).

**Conclusion:**

Compared with traditional systemic chemotherapy, docetaxel intraperitoneal infusion combined with chemotherapy has better therapeutic effect on gastric cancer ascites, with better survival benefit and tolerance and less hematological toxicity, which is worthy of further research and clinical application.

## Introduction

Gastric cancer with peritoneal metastasis is a special type of advanced gastric cancer with poor biological behavior and poor prognosis. In the patients with unresectable and recurrent gastric cancer, more than 50% had peritoneal metastasis during the clinical course, and the median survival time is only 4–6 months [1–3]. Besides, complications such as intestinal obstruction, abdominal infection, malnutrition, and cachexia caused by intractable malignant ascites also seriously affect the life quality of patients.

At present, chemotherapy is the main therapy for gastric cancer with peritoneal metastasis. Compared with cisplatin-5-fluorourasil (CF), a three-drug regimen such as docetaxel-cisplatin-5-fluorourasil (DCF) has a higher response rate (37% vs 25%) and longer survival benefit (9.2 vs 8.6 months) [4], which is more suitable for this type of disease with high malignant degree and poor prognosis. Despite great improvement in multimodal treatment, the prognosis remains poor for gastric cancer with peritoneal metastasis. One recent study reported that gastric cancer patients with peritoneal metastasis treated by systemic chemotherapy alone died within 6 months, indicating the ineffectiveness of systemic chemotherapy for peritoneal metastasis. Besides, the high hematological side effects of systemic chemotherapy also limit its scope of application.

Intraperitoneal chemotherapy is an important local treatment with which to improve the anti-tumor effect by increasing the concentration of local drugs in the abdominal cavity and slowing down the spread of chemotherapeutic drugs to plasma; it can also enter the liver through the portal vein to improve the therapeutic effect on liver and portal system tumor micrometastasis. In addition, after the elimination of the first-pass effect of drugs through the liver, it can greatly reduce the effect of systemic toxicity and improve the tolerance of patients. At present, it has been widely used in the treatment of abdominal metastasis of many kinds of solid tumors.

In this study, a chemotherapy regimen of intraperitoneal perfusion of docetaxel combined with SOX, oxaliplatin, and S-1 (an orally active combination of tegafur, gimeracil, and oteracil in a molar ratio of 1:0.4:1) was designed to further analyze the survival differences and side effects of docetaxel, oxaliplatin, and S-1 in advanced gastric cancer patients with malignant ascites under different administration modes.

## Methods

### Participants and study design

This study was conducted in accordance with the principles of Helsinki Declaration and Good Clinical Practice and was approved by the Institutional Review Boards of Guangxi Medical University Medical Center. Written informed consents were obtained from all patients.

Totally, 78 patients were enrolled in this study from 2016-1-20 to 2018-6-20. Through the stratified block randomization method, we made random number cards, sequentially numbered them into envelopes and kept them in the filing cabinet of the central office. After the patients were correctly screened by the research physicians, the central staff opened the envelopes in order and assigned them to the DOS group (control) and the D-SOX group. Inclusion criteria were as follows: participants (20–75 years old) histologically diagnosed as advanced gastric adenocarcinoma or esophagogastric junction adenocarcinoma, without prior palliative chemotherapy or radiotherapy, or with the first recurrence or metastasis 6 months or longer after neoadjuvant or adjuvant chemotherapy; patients had an estimated survival time > 3 months and Eastern Cooperative Oncology Group (ECOG) performance status of 0–2, with adequate cardio-pulmonary, hepatic, renal, and hematologic function; HER-2 (0 or 1+) or HER-2 (2+) and the HER-2 gene was not amplified by FISH test; cancer cells were found by ascites cytology; patients had measurable or non-measurable assessable lesions; initial treatment. Exclusion criteria were as follows: participants had intestinal obstruction, intestinal perforation or gastrointestinal bleeding, active infection, concurrent cancer, and brain or leptomeningeal involvement; patient declined to sign the informed consent or fail to comply with its requirements.

### Treatment schedule

Participants in the DOS group received intravenous oxaliplatin 100 mg/m^2^ (jiang Su Hengrui Pharmaceutical Co, Ltd) and docetaxel 60 mg/m^2^ (Shandong Qilu Pharmaceutical Co. Ltd.) on day 1, as well as oral administration of S-1 capsule (Jiangsu Hengrui Pharmaceutical Co. Ltd.) from day 1 to day 14. In the D-SOX group, patients were given intravenous oxaliplatin 100 mg/m^2^ on day 1, intraperitoneal perfusion of docetaxel 30 mg/m^2^ on day 1 and day 8, and oral administration of S-1 capsule from day 1 to day 14. The dosage of S-1 capsule was determined according to body surface area (< 1.25 m^2^, 40 mg twice a day; 1.25–1.5 m^2^, 50 mg twice a day; > 1.5 m^2^, 60 mg twice a day on days 1–14). Efficacy of both groups was evaluated every 2 cycles with 21 days as a cycle. Patients received protocol treatment until disease progression, occurrence of unacceptable toxicity, or patient withdrawal.

In intraperitoneal perfusion chemotherapy, drainage tubes were placed in both groups. In the 3–5 days before chemotherapy, 1000–2000 ml/day of peritoneal effusion was drained according to patient’s physique. Meanwhile, 1000–1500 ml saline containing dexamethasone 10 mg was perfused via intraperitoneal port or catheter, and the ascites was drained after several days of repetition. Intraperitoneal perfusion of docetaxel was given to the subjects in the D-SOX group. Firstly, 1000 ml of normal saline containing dexamethasone 10 mg and 2% lidocaine 5 ml was intraperitoneally injected through the drainage tube. After ensuring that patients did not feel ill, docetaxel 30 mg/m^2^ was diluted in normal saline 500 ml and administered intraperitoneally in 30–60 min. Finally, normal saline 500 mL was continuously infused through flushing pipes, and the abdomen was hot-compressed with a warm water bag after operation. Patients were advised to turn over regularly to change their positions, thereby promoting the uniform mixing of drugs in the abdominal cavity.

### Dose modifications

In the course of treatment, patients with progressive disease or unbearable severe adverse reactions to chemotherapy shall stop chemotherapy or switch to other chemotherapy regimens. When grade 3–4 non-hematological adverse reactions, grade 4 hematological adverse reactions, or febrile neutropenia occurred, the amount of chemotherapeutic drugs in the next cycle was reduced by 25%. What is more, the chemotherapy regimen should be stopped if the same or higher grade of toxic reaction still occurs in the second dose reduction or if the toxic reaction delayed the start of the preset cycle by more than 4 weeks.

### Response and toxicity evaluation

The primary endpoint was overall survival. The secondary endpoints were objective response rate (ORR), ascites control rate, negative conversion rate of ascites cytology, and side effects.

With reference to the response evaluation criteria in solid tumors guidelines (version 1.1), the efficacy evaluation of measurable lesions was divided into complete remission (CR), partial remission (PR), stable (SD), and progressive (PD). The response rate was defined as the proportion of patients with the best overall response of CR or PR. The volume of ascites was evaluated by ordinary computed tomography based on a 5-point measurement method [5]. According to the evaluation standard of WHO ascites, the curative effect was determined as follows: CR, celiac effusion disappeared and lasted for more than 4 weeks; PR, celiac effusion significantly decreased by more than 50% and lasted for more than 4 weeks; SD, peritoneal effusion decreased by less than 50% or increased by no more than 25%; PD, ascites increased by more than 25%. According to the National Cancer Institute Common Terminology Criteria for Adverse Events 4.0 (NCL-CTCAE v4.0), the adverse reactions were categorized as 1–5 grades.

Follow-up was conducted by outpatient, hospitalization, or telephone from the end of chemotherapy to the death or loss of follow-up. The last follow-up time was June 20, 2019.

### Statistical analysis

The primary objective of this study was to demonstrate that OS rate of patients treated with D-SOX was superior to that of patients treated with DOS. On the basis of previous research results [6, 7], we assume that the 1-year OS rate of D-SOX and DOS are 75% and 50%, respectively. According to Lachin-Foulkes method (PASS 11.0, NCSS, USA), one-sided log-rank test was adopted with *α* of 0.05, 90% power, and 5.0% loss, and the target sample size was set at 78 patients. SPSS software (version 19.0, IBM, USA) was used for statistical analysis. Pearson chi-square test or Fisher’s exact test was used to compare the counting data between groups. Kaplan-Meier survival curve was used for survival analysis. Log-rank test and Cox proportional hazards model were performed to compare survival time and estimate the risk ratio on the preset subgroup, respectively.

## Results

### Patients’ baseline data

According to the proportion of 1:1, the patients were randomly divided into observation group (D-SOX) and control group (*n* = 39), and stratified by ECOG performance status. As shown in Table 1, there were no significant differences in age, gender, degree of differentiation, previous chemotherapy, Lauren classification, ascites volume, or tumor burden between the two groups (*P* > 0.05).

**Table 1:** Baseline demographic and clinical characteristics

Characteristic	D-SOX (*n* = 39)	DOS (*n* = 39)	*P* value
Age (years)			
Median	50.5	50	
Range	33–68	35–65	
Sex			
Male	18 (46 %)	20 (51 %)	0.65
Female	21 (54 %)	19 (49 %)	
ECOG PS			
1	27 (69 %)	27 (69 %)	1.00
2	12 (31 %)	12 (31 %)	
Lauren classification			
Diffuse type	27 (69 %)	26 (67 %)	0.81
Mixed type	12 (31 %)	13 (33 %)	
Histologic type*			
Undifferentiated	31 (79 %)	28 (72 %)	0.43
Differentiated	8 (21 %)	11 (28 %)	
Previous chemotherapy			
Yes	9 (23 %)	6 (15 %)	0.39
No	30 (77 %)	33 (85 %)	
Amount of ascites**			
Moderate	23 (59 %)	28 (72 %)	0.23
Massive	16 (41 %)	11 (28 %)	
Burden of tumor***			
PM only	15 (38 %)	10 (28 %)	0.23
Compound PM	24 (62 %)	29 (74 %)	

Abbreviations: *ECOG PS* eastern Cooperative Oncology Group performance status, *PM* peritoneal metastasis; *Differentiated (well differentiated, moderately differentiated); undifferentiated, poorly differentiated adenocarcinoma (solid type, nonsolid type), signet ring cell carcinoma, and mucinous adenocarcinoma; ** Evaluated by computed tomography using a five-point method: Moderate, within the range of 1000–3000 ml; Massive, ascites beyond 3000 ml; *** PM only, peritoneal metastasis only, only abdominal implantation metastasis occurred; Compound PM, peritoneal metastasis combined with other metastases

### Comparison of primary and secondary outcomes between the DOS group and D-SOX group

**Fig. 1:**
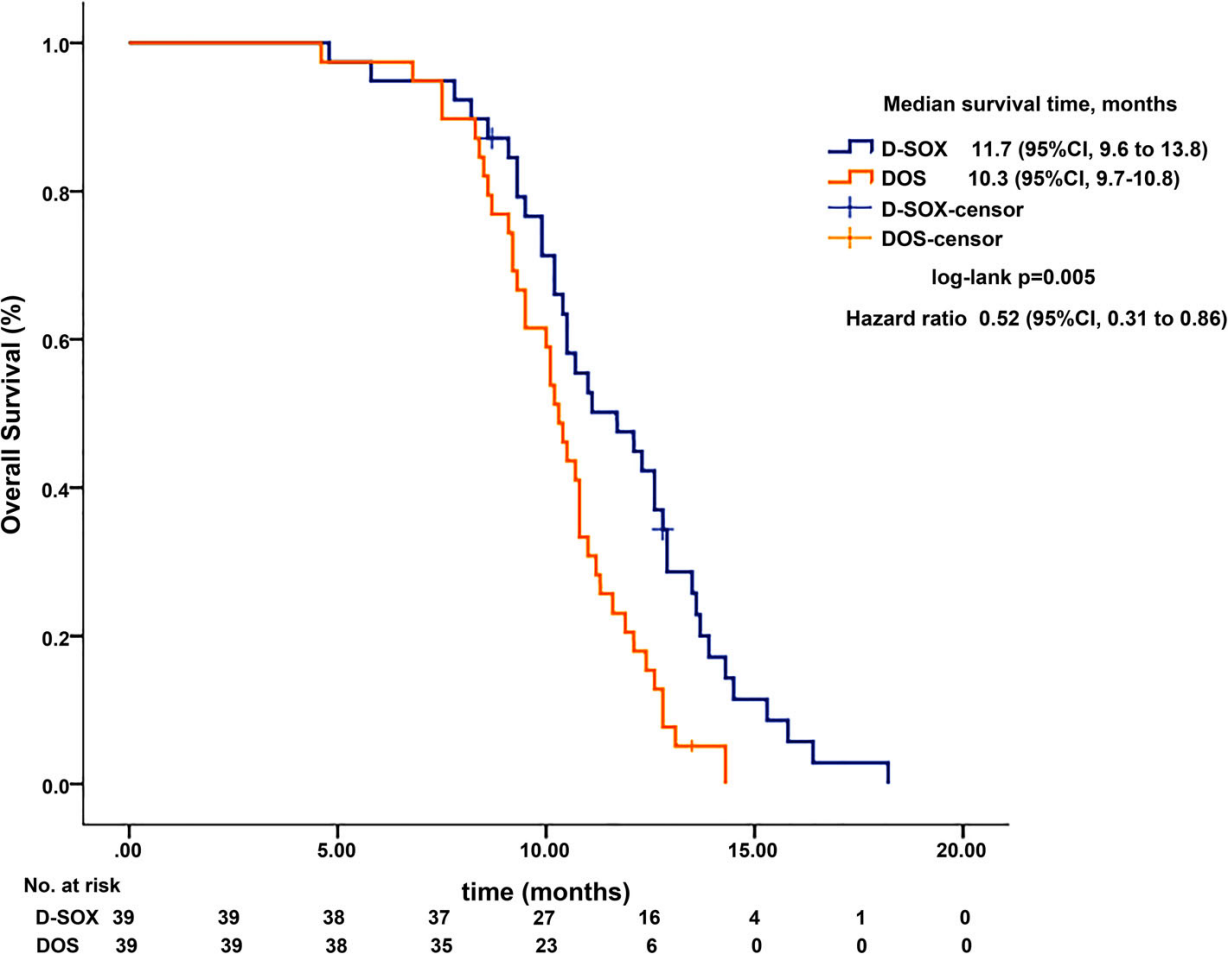
Overall survival by treatment arm (KM curve). D-SOX, intraperitoneal docetaxel and intravenous oxaliplatin plus taking orally S-1; DOS, intravenous docetaxel and oxaliplatin plus taking orally S-1

**Fig. 2:**
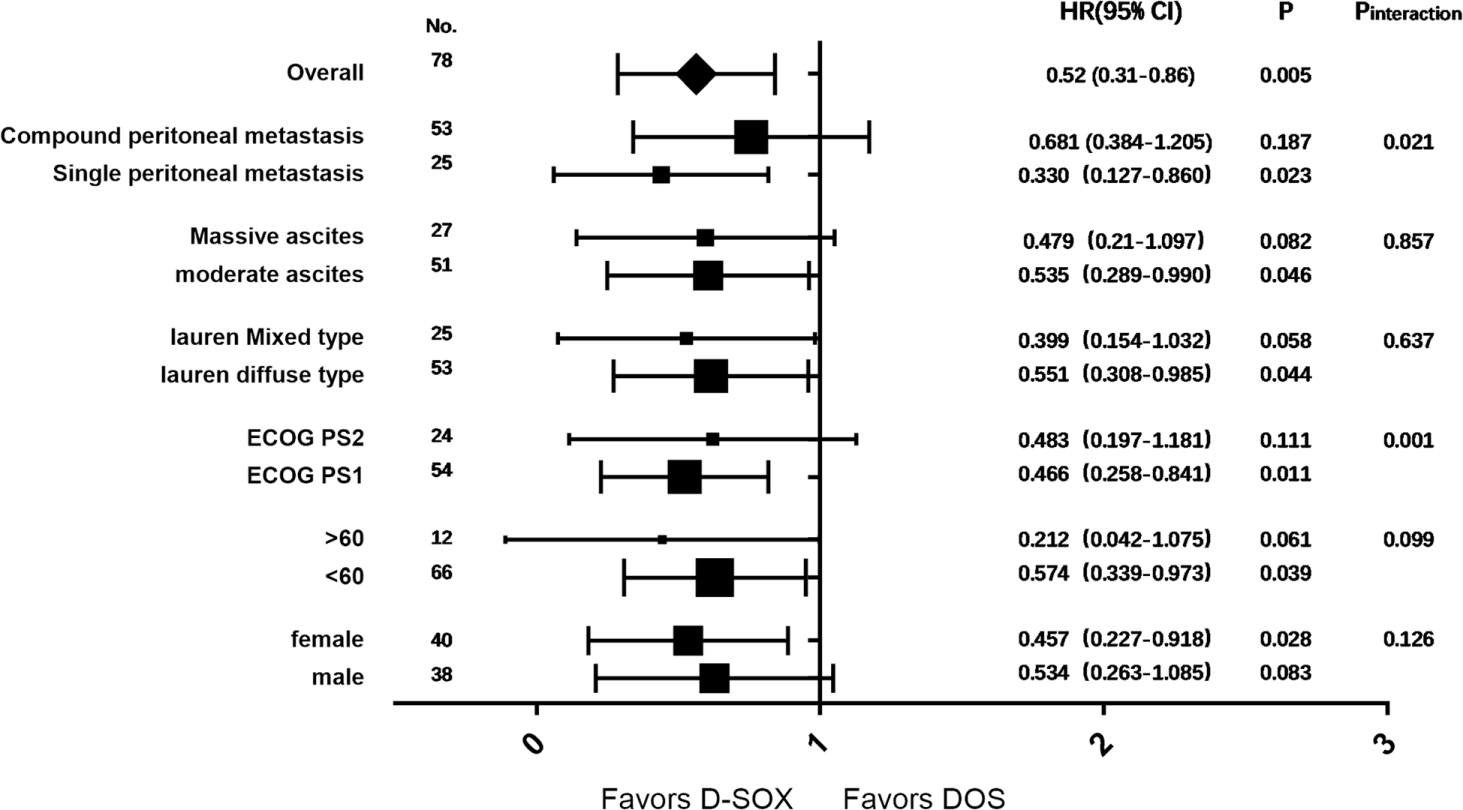
Subgroup analyses of overall survival. HR, hazard ratio; ECOG PS, Eastern Cooperative Oncology Group performance status; D-SOX, intraperitoneal docetaxel and intravenous oxaliplatin plus taking orally S-1; DOS, intravenous docetaxel and oxaliplatin plus taking orally S-1

All patients (D-SOX 39; DOS 39) received the allocated combination and, thus, comprised the full analysis population and were analyzed for efficacy and safety. There were 2 cases in the D-SOX group and 1 case in the DOS group shed off due to loss of follow-up, and no case was eliminated. The median OS was 11.7 months (95%CI 9.6–13.8) in the D-SOX group and 10.3 months (95%CI 9.7–10.8) in the DOS group. There was a statistically significant difference in OS between the two groups, with a hazard ratio of 0.52 (95%CI 0.31–0.86, *P* = 0.005) (Fig. 1). The ORR of the D-SOX group was 48.7% (19/39, 95%CI 32.3–65.1%) and that of the DOS group was 41.0% (16/39, 95%CI 24.9–57.2%), indicating no statistical significance between the two groups (*P* = 0.49). However, the ascites control rate of the D-SOX group was remarkably higher than that of the DOS group (58.9%, 95%CI 42.8–75.1% vs 30.8%, 95%CI 15.6–45.9%; *P* = 0.012) (Table 2). Besides, compared with the DOS group, D-SOX had longer median duration of ascites remission (5.6 ± 2.0 vs 4.4 ± 0.8 months, *t* ' = 2.397), a higher cytological negative conversion rate of ascites (69.4%, 95%CI, 53.6–85.3% vs 25%, 95%CI 10.1–39.9%), and less median number of negative conversion cycles (3.0 ± 0.8 cycles vs 4.3 ± 0.9 cycles, *t* = − 4.171) (*P* = 0.023, *P* < 0.001, and *P* < 0.001, respectively). The forest map subgroup analysis of overall survival showed that the interactions of treatment group with these factors were significant (*P* = 0.001 for ECOG PS and *P* = 0.021 for tumor burden). The effect of D-SOX on overall survival of patients with PS score of 1 or single peritoneal metastasis was greater than of patients with PS score of 2 or peritoneal metastasis combined with other metastases (*P* = 0.001 for ECOG PS and *P* = 0.021 for peritoneal metastasis) (Fig. 2).

**Table 2:** Response evaluation in the All-Patients-Treated Set

	Overall evaluation			Evaluation of ascites		
	PR	ORR(%)	*P*	PR	ORR(%)	*P*
D-SOX (*n* = 39)	19	48.7	0.49	23	58.9	0.012
DOS (*n* = 39)	16	41.0		12	30.8	

*ORR* objective response rate

### Complications in the two groups

Toxicity and side effects were evaluated in both arms, and there were no unexpected serious adverse events or treatment-related death. The most common grade 3/4 adverse events were leukopenia, neutropenia, anemia, and anorexia, with the DOS group having higher incidence of leukopenia (20% vs 46%, *P* = 0.02) and neutropenia (26% vs 54%, *P* = 0.01) than the D-SOX group. Moreover, the incidence of delayed chemotherapy due to hematologic adverse events was more frequent in the DOS arm (11.1% vs 33.3% *P* = 0.027). No significant differences between the two groups were found in terms of febrile neutropenia, abdominal pain, infection related to intraperitoneal ports, catheter obstruction, or tolerable nonhematologic toxicities including nausea, vomiting, anorexia, and peripheral neuritis (Table 3).

**Table 3:** Adverse events in the All-Patients-Treated Set

Adverse event	Grades 1–2			Grades 3–4		
	D-SOX (*n* = 39)%	DOS (*n* = 39)%	*P*	D-SOX (*n* = 39)%	DOS (*n* = 39)%	*P*
Leukopenia	23 (59%)	19 (49%)	0.36	8 (20%)	18 (46%)	0.02
Neutropenia	18 (46%)	16 (41%)	0.48	10 (26%)	21 (54%)	0.01
Anemia	30 (77%)	28 (72%)	0.60	5 (13%)	8 (21%)	0.36
Thrombocytopenia	12 (31%)	15 (38%)	0.47	2 (5%)	2 (5%)	1.00
Febrile neutropenia	–	–	–	1 (3%)	3 (8%)	0.61
Nausea	18 (46%)	21 (54%)	0.49	2 (5%)	4 (10%)	0.67
Vomiting	11 (28%)	16 (41%)	0.23	2 (5%)	3 (8%)	1.00
Diarrhea	15 (38%)	17 (44%)	0.65	2 (5%)	1 (3%)	1.00
Anorexia	25 (64%)	23 (59%)	0.64	4 (10%)	6 (15%)	0.5
Fatigue	22 (56%)	25 (64%)	0.48	2 (5%)	3 (8%)	1.00
AST increased	5 (13%)	8 (21%)	0.36	1 (3%)	1 (3%)	1.00
ALT increased	8 (21%)	10 (26%)	0.59	1 (3%)	1 (3%)	1.00
Creatinine increased	6 (15%)	8 (21%)	0.55	0	0	–
Peripheral sensory neuropathy	15 (38%)	19 (49%)	0.36	0	0	–
Rash maculopapular	3 (8%)	5 (13%)	0.71	0	0	–
Skin hyperpigmentation	15 (38%)	14 (36%)	0.82	0	0	–
Mucositis oral	13 (33%)	16 (41%)	0.48	0	0	–

*ALT* alanine transaminase, *AST* aspartate aminotransferase

## Discussion

Peritoneal implantation metastasis is the most typical metastasis mode in advanced diffuse gastric cancer, accompanied by increased peritoneal capillary permeability, lymphatic blockage, and lymphatic reflux disturbance, which are the main causes of carcinomatous ascites. Due to the existence of peritoneal-plasma barrier and disordered blood supply in peritoneal metastatic cancer, traditional chemotherapy drug cannot effectively infiltrate the peritoneal cavity. Therefore, it is urgent to find out an effective treatment for gastric cancer with cancerous ascites.

In recent years, intraperitoneal chemotherapy has been widely used because it not only increases the effective concentration of drugs in the cavity and enhances local efficacy, but also reduces the plasma exposure of drugs and the occurrence of systemic adverse reactions. A series of studies have shown that macromolecules, water solubility, easy ionization, and dissolution are the four main properties that affect the efficacy of intraperitoneal infusion chemotherapy [8–10]. It has been reported that taxanes have relatively high molecular weight and AUCIP/AUCplasma ratio, which can direct kill tumor cells and have more pharmacokinetic advantages than 5-Fu and DDP in intraperitoneal perfusion therapy. In addition, docetaxel, as a new generation of taxane drugs, is more water-soluble than ordinary paclitaxel, which can not only penetrate the tumor surface, but also be easily absorbed by peritoneal capillaries to play its dual cytotoxic effect [10].

However, owing to the limited tissue penetration capacity of chemotherapeutic drugs, the penetration depth of macromolecular substances such as taxanes is less than 100 μm [11], which may be the main reason for drug resistance of peritoneal cancer nodules fused into lumps. Therefore, systemic chemotherapy is equally important for advanced gastric cancer patients with peritoneal metastasis. Taxanes, fluorouracil and platinum are the three main chemotherapy drugs for advanced gastric cancer. As a new generation of oral fluorouracil, S-1 combined with cisplatin (SP regimen) achieved an objective effective rate of 54%, and the median overall survival reached 13 months [12], making it a new standard of first-line chemotherapy for advanced gastric cancer in East Asia. Oxaliplatin is the third generation platinum anticancer drug, which has synergistic effect with 5-FU and no cross-resistance with cisplatin. Meanwhile, its hematological toxicity is significantly less than cisplatin, making it widely applied in clinic. Yamagata S [13] confirmed that metastatic foci and adjacent fibrous connective tissues of cancerous ascites were rich in DPD enzyme. Compared with ordinary 5-FU, S-1 containing DPD enzyme inhibitor (CDHP) obtains higher drug concentration in abdominal cavity. The subgroup analysis of two other large phase III studies (START [14] and G-SOX [15]) also found that DS or SOX regimens tended to have better survival benefits in patients with histologically diffuse type. Therefore, docetaxel, S-1 and oxaliplatin, as the preferred drugs for diffuse gastric cancer with peritoneal metastasis, are worthy of further study.

Currently, the relationship between different drug administration modes and anti-tumor efficacy has become a new research direction. A Japanese PHOENIC-GC study in 2018 [16] suggested that there was no survival advantage for intraperitoneal and intravenous dual-channel administration compared with intravenous administration alone. Nevertheless, the imbalance between the study arms, especially the uneven distribution of ascites patients in the two groups, may be the main reason for the weakening of the advantage of the trial group. In this study, subjects with balanced clinical data, especially a large number of patients with cancerous ascites, were selected and treated with intraperitoneal infusion of docetaxel plus SOX regimen. Although no difference in the overall effective rate was observed between the two groups, the total survival time of the D-SOX group was longer than that of the DOS group. In addition, the ascites control rate, duration of ascites remission, and cytological negative conversion rate of ascites in the D-SOX group were significantly better than those in the DOS group. Therefore, as an important factor for poor prognosis of advanced gastric cancer, the control of malignant ascites affects the survival and overall therapeutic effect of patients to a great extent.

The three-drug combination regimen has a high effective rate, especially for patients with high tumor burden, refractory, and poor prognosis. However, the high-grade 3/4 hematological side effects limit its scope of application. Although the dose-adjusted DOS regimen has achieved a certain balance in terms of efficacy and toxicity [17, 18], hematological toxicity remains the main adverse event compared with the two-drug regimens. In this study, more than 50% of patients in the DOS group developed grade 4 neutropenia, compared with 28% of patients in the D-SOX group. Therefore, the D-SOX group had better treatment compliance and lower incidence of delayed chemotherapy. At present, the administration mode of taxanes varies from study to study, resulting in different adverse events. The incidence of grade 3/4 neutropenia in combined therapy of intraperitoneal perfusion and intravenous administration was 20–68% [19–21], compared with 5.5 to 7.4% in single intraperitoneal chemotherapy [22, 23]. No adverse reactions such as grade 3/4 abdominal pain were reported, except for one study of up to 37.5% [21], in which intraperitoneal perfusion with docetaxel and cisplatin was performed simultaneously. In our study, there were no intraperitoneal chemotherapy-related complications, such as abdominal pain, intestinal obstruction, and infectious peritonitis.

In spite of not having a significant difference in ORR between the two arms, the D-SOX group had great advantages in ascites control rate and ascites cytological negative conversion rate, which effectively suppressed the important factors of poor prognosis of advanced gastric cancer, increased overall survival time, and improved life quality of patients. This confirmed the efficacy of dual chemotherapy mode of systemic combined with local intraperitoneal perfusion, which can better exploit the advantage of dual cytotoxicity of docetaxel, while avoiding the deficiency of hematological toxicity of the three drugs, which is more in line with the consideration of East Asian population individualization.

In conclusion, D-SOX regimen has more significant effects in terms of survival benefit, tolerance, and hematological toxicity when compared with traditional systemic chemotherapy. Therefore, D-SOX regimen can be used as a potent treatment for advanced gastric cancer patients with peritoneal metastasis in the absence of effective treatment. But it is worth noting that the localized separation and encapsulation of ascites is an important factor weakening the effect of chemotherapy perfusion; the combined application of thermal perfusion may be a new research direction to further improve the efficiency of this administration mode.

## Data Availability

The datasets used and/or analyzed during the current study are available from the corresponding author on reasonable request.

## Abbreviations

CF: Cisplatin-5-fluorourasil
DCF: Docetaxel-cisplatin-5-fluorourasil
ECOG: Eastern Cooperative Oncology Group
ORR: Objective response rate
CR: Complete remission
PR: Partial remission
SD: Stable
PD: Progressive
